# Stethoscope hygiene: A legal consideration for cardiologists practicing in a new era of infection control (COVID-19)

**DOI:** 10.1016/j.ahjo.2021.100039

**Published:** 2021-07-30

**Authors:** Rajiv S. Vasudevan, Alpesh Amin, Daniel L. Hannula, Alan S. Maisel

**Affiliations:** a Department of Medicine, University of California, San Diego School of Medicine, La Jolla, CA, United States of America; b Department of Medicine, University of California, Irvine School of Medicine, Irvine, CA, United States of America; c Rush, Hannula, Harkins, Kyler LLP, Tacoma, WA, United States of America

**Keywords:** Stethoscope, Infection, Malpractice, Clinical practice, COVID-19

## Abstract

The stethoscope is a tool cherished by the field of cardiology and ubiquitous throughout medicine. However, little consideration has been given to its safe usage regarding its potential for pathogenic contamination despite thorough evidence that stethoscopes can harbor pathogens that can be transmitted to patients upon contact. The COVID-19 (SARS-COV-2) pandemic has led to increased infection control vigilance, including toward the stethoscope, as evidenced by a recent increase in literature highlighting stethoscope hygiene/contamination. A consequence of this increase in awareness is that stethoscopes may be implicated in medical malpractice lawsuits as a potential cause of healthcare-associated infections (HAIs). While there is limited evidence demonstrating a direct connection between stethoscope contamination and HAIs, malpractice lawsuits often do not require direct causative evidence. Regardless, efforts should be made to bolster stethoscope hygiene to not only mitigate patient harm, but also prevent providers from potential medical-legal conflicts. The continued relevance and utility of the stethoscope as a rapid, cost-effective diagnostic tool needs to be appropriately balanced with increased hygiene performance. Providers should anticipate increased scientific evidence and patient awareness regarding stethoscope contamination in the post-COVID-19 era.

## 1 Background

The stethoscope has long been at the center of the physical examination as both an accessible diagnostic tool as well as a symbol of the art of medicine. Cardiology remains as among the medical disciplines that continues to highly regard the stethoscope as a rapid, cost-effective, and informative tool that can be used in any medical setting [1]. Despite its ubiquity among cardiologists and medicine overall, few considerations have been given to its safe usage. This notion has been largely ignored until recently; the COVID-19 (SARS-COV-2) pandemic has ushered in a new era of infection control vigilance, and stethoscope contamination has recently gained attention as poorly addressed sector of infection control with implications for the spread of COVID-19 [2]. A case report published in the *European Heart Journal* implicates a stethoscope in potentially transmitting COVID-19 to a provider who was auscultating a patient with active infection [3]. A recent commentary in a Centers for Disease Control and Prevention advocacy journal called for updated stethoscope hygiene guidelines to appropriately address the risk posed by a contaminated stethoscope [4]. While there lacks sufficient evidence to demonstrate that the COVID-19 virus can contaminate and be transmitted from a stethoscope, it is likely that there has been an increase in awareness regarding stethoscope contamination among providers and patients. Thus, considering that stethoscopes likely contribute to spreading infection in healthcare settings, not performing stethoscope hygiene may be the basis for medical malpractice lawsuits. However, there is an ideological gap between the danger posed by the stethoscope from bacterial contamination and the current state of vigilance toward this potential danger; put simply, many physicians are not aware that failure to perform stethoscope hygiene could potentially constitute negligence.

Thus, the purpose of this article is to emphasize that the medical community must first bolster its awareness and response in order to primarily protect patients from healthcare-associated infections; and second take prophylactic measure against the potential legal ramifications that might ensue if a contaminated stethoscope is implicated.

## 2 Defining HAI malpractice

Healthcare-associated infections (HAIs), or infections acquired while receiving care in a healthcare setting, are a significant cause of morbidity and mortality in the United States. According to a 2011 report by the Centers for Disease Control and Prevention (CDC), an estimated 721,000 HAIs occurred in acute care settings, with approximately 75,000 deaths as a result [5]. Furthermore, HAIs had a direct attributable cost between 25 and 48 billion dollars annually on the healthcare system [6]. However, indirect costs, such as costs of litigation and plaintiffs’ verdicts, are also a significant source of expenditure for healthcare organizations and providers. One study found that the medical legal system had an annual expenditure of 55.64 billion dollars (2008). This cost consists of indemnity payments, defendant/plaintiff fees, and administrative and overhead costs [7]. The basis for malpractice litigation often involves negligence in providing substandard medical care for disease recognition and management. This can include failing to order proper testing, not making a proper and timely diagnosis, performing an unnecessary procedure/surgery, or neglecting to perform certain safety/prophylactic measures intended to mitigate harm to patients [8]. Ultimately, harm to the patient by any of the above means can be grounds for a malpractice lawsuit.

In the realm of HAIs, performance of proper hygiene is the main deterrent to patient harm [9], and thus can be subject to legal ramifications if neglected. While it can be difficult to identify the causative source of a healthcare-acquired infection, a healthcare facility can be liable if the infection was contracted during a patient's care and expected hygiene measures were not performed [10]. For example, hand sanitizing is one of the most emphasized facets of healthcare hygiene practices, and its longstanding emphasis since the late 19th century [11] has led to established guidelines for proper hand hygiene in healthcare settings [12]. Despite this, hand hygiene continues to be underperformed, with studies demonstrating poor hand hygiene compliance in both ICU (40-50%) and non-ICU (50-60%) settings [13]. Studies have also demonstrated that poor hand hygiene alone can result in healthcare expenditures of approximately $50,000 per infected patient [14], and is associated with increased mortality and length of hospital stay [15]. Thus, given that hands are the most notable vectors for infection and are thus governed by strict hygiene guidelines, it is not surprising that poor hand hygiene has been implicated in HAI lawsuits [16].

**Fig. 1:**
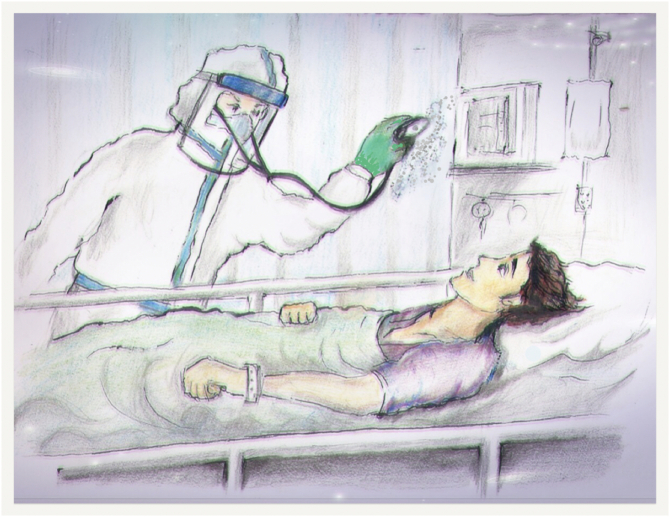
Neglecting stethoscope hygiene. Artistic dramatization of the current state of stethoscope hygiene awareness. The provider, pictured standing over the patient, takes the appropriate barrier precaution for a patient who is under contact precautions, but neglects to consider that his/her stethoscope may be contaminated prior to auscultating the patient. The patient awaits this commonplace and familiar aspect of the physical exam, unknowing to both patient and provider that the stethoscope may be contaminated and could colonize the patient upon contact.

While hand hygiene in addition to other forms of antimicrobial and barrier precaution have been emphasized (e.g., wearing a mask, gloves, gown, face shield etc.), little attention has been given to the stethoscope– an instrument in medicine that comes in frequent contact with patients, perhaps second only to a provider's hands (Fig. 1). Prior studies have demonstrated that stethoscopes can be contaminated to the same extent as a provider's hand [17], with bacteria on a stethoscope being transferable to patients upon contact [18]. Despite these findings, stethoscope hygiene rates remain low according to both survey-based and observational studies [19], [20], [21], [22]. Furthermore, guidelines from the CDC on stethoscope hygiene are ambiguous; stethoscopes are classified as non-critical medical devices, and cleaning is “recommended” anywhere between after each patient encounter to once a week [23]. Despite the stethoscope being appropriately coined the “third-hand” of the physician with regard to its symbolism, utility, and potential for contamination [24], hygiene measures are highly deficient and lack uniformity.

## 3 HAI malpractice cases

HAIs resulting from alleged hygiene negligence is a relatively common cause for medical malpractice lawsuits [25]. However, it is important to note that there is a level of infection risk that is associated with healthcare that is difficult to avoid. For example, surgical procedures carry a 3-4% risk of infection even when all of the necessary hygienic precautions are taken [26]. In fact, one study from the Harvard School of Public Health found that only 27% of adverse medical events are due to negligence [27]. Limiting healthcare-acquired infection is often more difficult than other forms of adverse event prevention due to the elusiveness of contaminated surfaces in healthcare environments, myriad of infection sources, and variability of host defense capability. Thus, the current guidelines on infection prevention have focused on mediators of infection that can be reasonably controlled (e.g., hand washing/sanitizing before and after physical examination, sterilization of medical/surgical equipment, & maintaining proper contact precautions for immunocompromised individuals). Therefore, hygiene-negligent malpractice often implies that a provider failed to meet the medical standard expected of hygienic practice.

Poor disinfection/sterilization of medical devices, instruments, and surfaces are often cited as justification for HAI-associated malpractice (Table 1). A typical case presentation involves the plaintiff claiming that an infection they developed was the result of a procedure that was performed in a healthcare setting. Table 1 summarizes examples of HAI lawsuits involving a claim against a healthcare entity for causing an infection by methicillin-resistant *Staphylococcus aureus* (MRSA) [28], [29], [30], [31]; the cases illustrated do not involve a stethoscope, as there is yet to be a documented HAI lawsuit involving a contaminated stethoscope. The items often implicated in HAI lawsuits are things that have been established as vectors for infection (e.g., needles, surgical instruments, indwelling catheters, venous/arterial lines). However, a definitive source of infection doesn't always need to be cited to substantiate a case. For example, *Smith* vs. *USA* involved the plaintiff claiming to have contracted a MRSA infection while receiving care at a federally funded healthcare facility without naming the specific infection source [29]. Furthermore, one review of medical malpractice cases found that lawsuits can even be substantiated without medical evidence/documentation of infection, but rather just the verbal/written testimony of the plaintiff [25]. The ambiguity that can surround finding a causative source of infection lends to broad interpretations of how infection can be transmitted. Therefore, it is not unreasonable to predict that stethoscopes, which are ubiquitous in healthcare facilities, come in frequent contact with patients, and can be contaminated with infectious pathogens, could be implicated in a HAI lawsuit.

**Table 1:** Example medical malpractice lawsuit cases involving methicillin-resistant *Staphylococcus aureus* transmission. The above cases are provided as examples of HAI malpractice cases to demonstrate typical rationales for alleged infection-related medical malpractice. Rationales range from explicit procedural hazards to nonspecific exposures while receiving care at a medical facility.

Case (year)	Summary of claim	Implicated route of infection	Settlement amount
*Zangara* vs. *Advocate Health & Hospitals Corp.* (2017) [28]	*Plaintiff alleges that hospital failed to maintain a sterile environment and decolonize the plaintiff prior to performing coronary artery bypass, which resulted in an MRSA infection.*	Lack of sterile environment in operating room and improper decolonization of plaintiff.	$300,000
*Smith* vs. *USA* (2018) [29]	*Plaintiff claims that he was improperly treated for an infection on the back of his neck, and contracted MRSA while being seen at a federally funded medical clinic.*	Nonspecific exposure during visit	$12,647,009
*Cousin* vs. *River West Medical Center* (2013) [30]	*Patient received a shoulder injection following a work-related shoulder injury, resulting in MRSA tricuspid valve endocarditis, bacteremia, sepsis, and lung abscess. Plaintiff claims that the needle was not properly sterilized, and a sterile medical environment was not maintained.*	Shoulder injection from contaminated needle.	$509,705
*Alloway* vs. *Morrison* (2014) [31]	*Swelling and discoloration around PICC line following colorectal surgery, which was caused by MRSA infection. Plaintiff later was hospitalized for mitral valve colonization and perforation. Plaintiff claims that the surgeon failed to properly examine the PICC insertion site prior to discharge.*	Infection from indwelling catheter.	$847,410

Case files were retrieved from the LexisNexis™ legal document database.

## 4 Causation between stethoscopes and infection

Notable considerations in proving the legal culpability of stethoscope hygiene are 2-fold: first, establishing negligence for failure to properly prevent stethoscope contamination; and second, to prove a causal connection between the negligence and the harm to the patient caused by the transfer of pathogens from the stethoscope to the patient. Notably, there has yet to be a documented legal case where a contaminated stethoscope (determined via search query in LexisNexis™ legal database) was found to be the source of infection. An explanation for this could be that patients are not yet aware of stethoscope contamination and defer to other potential causative sources of infection. Another explanation could be that stethoscope contamination simply does not confer the same transfer risk as other more invasive medical devices.

One of the most contentious topics within stethoscope contamination is a lack of thorough evidence proving that a contaminated stethoscope caused a HAI. It has been proven that stethoscopes can be contaminated with a variety of pathogens, including MRSA [32], [33], [34], and those pathogens can be transferred to skin upon contact [18]; however evidence that a stethoscope has led to an infection or infectious outbreak is sparse in the literature. An early letter to the editor in *JAMA* from Garner et al. (1982) investigated stethoscopes as a potential vector during a hospital outbreak of methicillin and tobramycin-resistant *Staphylococcus aureus.* Out of 32 provider stethoscopes that were cultured, one was found to be contaminated with methicillin and tobramycin-resistant *Staphylococcus aureus* [35]*.* Another study investigated environmental contamination during an outbreak of *Klebsiella pneumoniae* in a neonatal intensive care unit (NICU)*.* The infection-causing strain of *K. pneumoniae* was discovered on several surfaces, including two incubator-specific stethoscopes and a provider stethoscope [36]. A more recent case report (2015) claimed via root cause analysis that a stethoscope transmitted *Listeria monocytogenes* between neonates due to a lack of cleaning [37].

While these findings suggest a causative role of the stethoscope in transmitting infectious disease, it is difficult to make a definitive claim regarding a relationship between stethoscope contamination and HAI. Disease-causing microbes can often be quite elusive and fastidious, contaminating a variety of surfaces and surviving on those surfaces for an extended period of time [38]. Thus, causation is difficult to prove with regard to singular vectors, such as a stethoscope. However, the elusiveness of pathogens and contaminated sources is an intrinsic facet of infection control that is difficult to avoid; but as evidenced by HAI lawsuit mechanisms of injury, the level of proof regarding infection transmission does not necessarily need to demonstrate a causative relationship between vector and recipient– only that precautions against the *possibility* of transmission were not taken (Table 1). With regard to the stethoscope, it simply might be enough to claim that because stethoscope hygiene was not performed, as implicated by the studies mentioned above, a HAI resulted from negligent hygiene practices.

## 5 Awareness of stethoscope contamination

Stethoscope contamination was first reported in the literature in the 1970s– roughly 50 years ago [39]. Despite this there has been poor awareness and action toward stethoscope hygiene, evidenced by a lack of hygiene practice [19], [20] and lack of specific guidelines for stethoscope hygiene [40]. However, in recent years, numerous studies and review articles have been published characterizing stethoscope hygiene. In 2019, *The New England Journal of Medicine* published a “Journal Watch,” commentary on stethoscope hygiene, claiming that stethoscope hygiene should be emphasized to the same extent as handwashing [41].

COVID-19 has intensified infection control vigilance in both the medical community and the public at large. The CDC has well-defined guidelines regarding the proper usage of personal protective equipment and hygiene [42], but recommendations regarding the stethoscope have been neglected. Given that the stethoscope might have utility in the diagnosis of COVID-19 multifocal pneumonia in resource-bereft settings, it is not surprising that several articles have been recently published addressing stethoscopes during COVID-19, highlighting both its utility as a diagnostic tool [43] and its potential danger as a vector for infectious disease [2], [3], [43], [44], [45]. In addition to revolutionizing our approach to infection control, COVID-19 seems to have also catalyzed awareness regarding stethoscope hygiene.

While stethoscope contamination is gaining traction in the medical community, it appears to be garnering a focus in the world of malpractice litigation. An online search with the key words “stethoscope, infection, malpractice” yields dozens of results with advertisements from law firms, seeking clients who claim that they suffered an infection from a contaminated stethoscope [46], including large firms who specialize in mass tort litigation against medical device makers. It is clear that stethoscope contamination is gaining awareness, and patients and the public at large are hearing that there is ‘more than meets the eye’ when it comes to the stethoscope. It is essential that the medical community “catch-up” to this shift in perception and attitudes and appropriately address stethoscope hygiene in order to keep patients safe and prevent malpractice litigation if we fail to do so.

## 6 Mitigating legal risk and optimizing quality: solutions for stethoscope hygiene

Limiting the harm associated with stethoscope hygiene requires defining “proper practice” for stethoscope hygiene, which lacks a strict definition according to current CDC guidelines/recommendations [40]. Thus, as it stands, there is no defined “standard of care” that can be referenced if a stethoscope is implicated in a lawsuit, other than the nonspecific recommendations for “non-critical” devices. We, the authors, recommend that stethoscope hygiene guidelines should be made more specific, recommending disinfection before and after each patient encounter to reflect the similarity that the stethoscope shares with the hand in terms of frequency of contact and level of contamination [17]. This would not only protect patients from the various nosocomial pathogens that could be present on the stethoscope, but also establish a standard of care for providers to cite in the event of a HAI allegation.

However, while physicians might be aware that stethoscopes can be contaminated, practices are highly deficient [47]. Providers cite a lack of time, poor access to cleaning supplies, and forgetfulness as reasons for not performing stethoscope hygiene [47]. Thus, it is clear that in order to keep patients safe and prevent malpractice, there needs to be a foundation of education and efficient methodology in performing stethoscope hygiene. Unfortunately, prior studies utilizing educational interventions have been met with poor success. A study by Holleck et al. found that an educational intervention involving informational sessions, distribution of cleaning supplies, and posted reminders did not improve observed stethoscope hygiene rates [21]. A recent study by Holleck et al. utilized an intervention where bioluminescent markers were used to visually demonstrate stethoscope contamination to providers. The study improved beliefs that stethoscopes could be contaminated by pathogens but failed to improve stethoscope hygiene rates [22].

The “resistance” to perform stethoscope hygiene not only highlights our practice deficiencies, but also may be conducive to unsafe stethoscope hygiene practices. Given the nature of high-workflow healthcare settings, it can be easy to forgo stethoscope hygiene when there are barriers in education, efficiency, and methodology. Stethoscope hygiene is one of the few realms of infection control that has not been subject to significant innovation, let alone any significant focus from the medical community. However, recent developments in stethoscope hygiene technology might have the potential to subvert the barriers to stethoscope hygiene. Among these innovations include antimicrobial copper-based stethoscopes [48], a UV-light stethoscope diaphragm case [49], and a touch-free stethoscope diaphragm cover dispenser [43]. As the landscape of stethoscope hygiene technology evolves with an increasing awareness of stethoscope contamination, we hope that stethoscope hygiene becomes ubiquitous in healthcare to bolster patient safety and provider accountability.

## 7 Takeaways for the contemporary cardiologist

Modern medicine is wrought with ever-increasing demands from patient care, administration, and the medical-legal sector. These shifting demands in medicine have led to a reappraisal of the stethoscope's role, where some have advocated for its obsoletion in lieu of other more technically advanced technologies, such as point-of-care ultrasound (POCUS) [50]. However, even proponents of POCUS acknowledge that its integration in the armamentarium of a physician will require extensive training beginning at the early stages of medical training, and that improper training can lead to misdiagnoses [50]. Furthermore, there is growing concern regarding the increased reliance on advanced technology compromising bedside manner [51]. The stethoscope, the proper usage of which is deeply embedded in medical education at all stages, continues to be a highly informative, rapid, and inexpensive diagnostic tool capable of detecting several cardiopulmonary abnormalities. Examples include presence of an S3 heart sound, which is highly predictive of left ventricular dysfunction, pericarditis without effusion, and pulmonary hypertension [1].

The stethoscope will continue to be an integral tool for all physicians, especially for cardiovascular medicine, despite the challenges the stethoscope faces from contamination and the potential medical-legal implications. Performance of stethoscope hygiene before and after each patient encounter, conventionally by the application of an alcohol-based wipe/pad, is the most ideal action to keep patients safe and prevent legal ramifications. The time-intensive nature of ideal stethoscope hygiene is a notable obstacle for many physicians [47]; therefore, while physicians should take the most comprehensive approach against stethoscope contamination, innovation in hygiene technology and methodology shows promise in making stethoscope hygiene efficient while maintaining efficacy [43].

## 8 Conclusion

The medical and legal world share an ethical obligation to protect the best interests of the public, whether it be through providing care that improves patient health and well-being as a physician or enforcing the justice system as a legal professional. Thus, as stethoscope contamination garners awareness as a potential vector for infectious diseases, both the medical and legal community will have a shared impetus to ensure that patients are being protected against harm to the greatest achievable level. Appropriate measures, such as specific guidelines from the CDC or interventions to improve stethoscope hygiene, should be taken, not only to ensure that we are protecting the health and safety of our patients, but to make providers aware and accountable for the potential danger posed by the stethoscope.

## Funding

None.

## Declaration of competing interest

**RSV** has no conflicts of interest to disclose.

**AA** reported serving as PI or co-I of clinical trials sponsored by NIH/NIAID, NeuroRx Pharma, Pulmotect, Blade Therapeutics, Novartis, Takeda, Humanigen, Eli Lilly, PTC Therapeutics, OctaPharma, Fulcrum Therapeutics, Alexion. He has served as speaker or consultant for BMS, Pfizer, BI, Portola, Sunovion, Mylan, Salix, Alexion, AstraZeneca, Nabriva, Paratek, Bayer, Tetraphase, Achogen La Jolla, Millenium, Ferring, PeraHealth, HeartRite, Aseptiscope, Sprightly.

**DLH** is a partner at Rush, Hannula, Harkins, Kyler LLP, a personal injury and medical malpractice law firm.

**ASM** is a Founder and Chief Clinical Officer of Aseptiscope Inc.

## References

[1] Fuster V. (2016). The stethoscope’s prognosis very much alive and very necessary. J. Am. Coll. Cardiol..

[2] Buonsenso D., Pata D., Chiaretti A. (2020). COVID-19 outbreak: less stethoscope, more ultrasound. Lancet Respir. Med..

[3] Vasudevan R.S., Bin Thani K., Aljawder D. (2020). The stethoscope: a potential vector for COVID-19?. Eur. Heart J..

[4] Kalra S., Amin A., Albert N. (2021). Stethoscope hygiene: a call to action. Recommendations to update the CDC guidelines. Infect. Control Hosp. Epidemiol..

[5] Magill S.S., Edwards J.R., Bamberg W. (2014). Multistate point-prevalence survey of health care-associated infections. N. Engl. J. Med..

[6] Scott R.D. (2009). The direct medical costs of healthcare-associated infections in U.S. hospitals and the benefits of prevention. Cdc. http://www.cdc.gov/hai/pdfs/hai/scott_costpaper.pdf.

[7] Mello M.M., Chandra A., Gawande A.A. (2010). National costs of the medical liability system. Health Aff..

[8] Bal B.S. (2009). An introduction to medical malpractice in the United States. Clin. Orthop. Relat. Res..

[9] Saint S., Kowalski C.P., Banaszak-Holl J. (2010). The importance of leadership in preventing healthcare-associated infection: results of a multisite qualitative study. Infect. Control Hosp. Epidemiol..

[10] Barnes B.A. (2010). Negligence, medical malpractice, vicarious liability, or patient responsibility: who should pay when a patient contracts MRSA from a healthcare facility?. Indiana Health Law Rev..

[11] Best M., Neuhauser D. (2004). Ignaz Semmelweis and the birth of infection control. Qual. Saf. Heal. Care..

[12] Sehulster L., Chinn R.Y. (2003). CDC; HICPAC. Guidelines for environmental infection control in health-care facilities. Recommendations of CDC and the Healthcare Infection Control Practices Advisory Committee (HICPAC). MMWR Recomm Rep..

[13] Erasmus V., Daha T.J., Brug H. (2010). Systematic review of studies on compliance with hand hygiene guidelines in hospital care. Infect. Control Hosp. Epidemiol..

[14] Cummings K.L., Anderson D.J., Kaye K.S. (2010). Hand hygiene noncompliance and the cost of hospital-acquired methicillin-resistant Staphylococcus aureus infection. Infect. Control Hosp. Epidemiol..

[15] Harris B.D., Hanson C., Christy C. (2011). Strict hand hygiene and other practices shortened stays and cut costs and mortality in a pediatric intensive care unit. Health Aff..

[16] Can Gallese P. (2019).

[17] Longtin Y., Schneider A., Tschopp C. (2014). Contamination of stethoscopes and physicians’ hands after a physical examination. Mayo Clin. Proc..

[18] Marinella M.A., Pierson C., Chenoweth C. (1997). The stethoscope: a potential source of nosocomial infection?. Arch. Intern. Med..

[19] Vasudevan R.S., Mojaver S., Chang K.W. (2019). Observation of stethoscope sanitation practices in an emergency department setting. Am. J. Infect. Control.

[20] Boulée D., Kalra S., Haddock A. (2019). Contemporary stethoscope cleaning practices: what we haven’t learned in 150 years. Am. J. Infect. Control.

[21] Holleck J.L., Merchant N., Lin S. (2017). Can education influence stethoscope hygiene?. Am. J. Infect. Control.

[22] Holleck J.L., Campbell S., Alrawili H. (2020). Stethoscope hygiene: using cultures and real-time feedback with bioluminescence-based adenosine triphosphate technology to change behavior. Am. J. Infect. Control.

[23] Rutala W.A., Weber D.J. (2008). Guideline for disinfection and sterilization in healthcare facilities. https://stacks.cdc.gov/view/cdc/47378.

[24] Jenkins I.H., Monash B., Wu J. (2015). The third hand: low rates of stethoscope hygiene on general medical services. J. Hosp. Med..

[25] Studdert D.M., Mello M.M., Gawande A.A. (2006). Claims, errors, and compensation payments in medical malpractice litigation. N. Engl. J. Med..

[26] Sohn D.H. (2013). Negligence, genuine error, and litigation. Int. J. Gen. Med..

[27] Brennan T.A., Leape L.L., Laird N.M. (2004). Incidence of adverse events and negligence in hospitalized patients: results of the Harvard Medical Practice Study I. 1991. Qual. Saf. Health Care.

[28] Zangara V. (2011).

[29] Smith V. (2018).

[30] Cousin V., River W. (2013).

[31] Alloway Noel Kevin, Alloway Linda J. (2014).

[32] Fenelon L., Holcroft L., Waters N. (2009). Contamination of stethoscopes with MRSA and current disinfection practices. J. Hosp. Infect..

[33] Hill C., King T., Day R. (2006). A strategy to reduce MRSA colonization of stethoscopes [9]. J. Hosp. Infect..

[34] Alali S.A., Shrestha E., Kansakar A.R. (2020). Community hospital stethoscope cleaning practices and contamination rates. Am. J. Infect. Control.

[35] Garner T.K., Rimland D. (1982). Stethoscopes and infection. JAMA J. Am. Med. Assoc..

[36] Gastmeier P., Groneberg K., Weist K. (2003). A cluster of nosocomial Klebsiella pneumoniae bloodstream infections in a neonatal intensive care department: identification of transmission and intervention. Am. J. Infect. Control.

[37] Fullerton L., Norrish G., Wedderburn C.J. (2015). Nosocomial neonatal listeria monocytogenes transmission by stethoscope. Pediatr. Infect. Dis. J..

[38] Bean B., Moore B.M., Sterner B. (1982). Survival of influenza viruses on environmental surfaces. J. Infect. Dis..

[39] Gerken A., Cavanagh S., Winner H.I. (1972). Infection Hazard from stethoscopes in hospital. Lancet Infect. Dis..

[40] Rutala W.A., Weber D.J. (2017). HICPAC HICPAC. Guideline for disinfection and sterilization in healthcare facilities. Cent. Dis. Control Prot..

[41] Ellison R. (2019). Stethoscope contamination. N. Engl. J. Med..

[42] Interim infection prevention and control recommendations for patients with confirmed coronavirus disease 2019 (COVID-19) or persons under investigation for COVID-19 in healthcare settings. Centers Dis. Control Prev.

[43] Vasudevan R.S., Horiuchi Y., Torriani F.J. (2020). Persistent value of the stethoscope in the age of COVID-19. Am. J. Med..

[44] Marinella M.A. (2020). COVID-19 pandemic and the stethoscope: do not forget to sanitize. Hear. Lung..

[45] Zhu J., Tan Y., Huang B., Zhu Y., Gao XH. (2021). Don't throw the stethoscope away!. Eur. Heart J..

[46] Stethoscope, Infection, Malpractice. https://www.google.com/search?q=Stethoscope%2C Infection%2C Malpractice&oq=Stethoscope%2C Infection%2C Malpractice&aqs=chrome..69i57j33i160l3j33i299.5389j0j7&sourceid=chrome&ie=UTF-8.

[47] Muniz J., Sethi R.K.V., Zaghi J. (2012). Predictors of stethoscope disinfection among pediatric health care providers. Am. J. Infect. Control.

[48] Schmidt M.G., Tuuri R.E., Dharsee A. (2017). Antimicrobial copper alloys decreased bacteria on stethoscope surfaces. Am. J. Infect. Control.

[49] Messina G., Burgassi S., Messina D. (2015). A new UV-LED device for automatic disinfection of stethoscope membranes. Am. J. Infect. Control.

[50] Solomon S.D., Saldana F. (2014). Point-of-care ultrasound in medical education - stop listening and look. N. Engl. J. Med..

[51] Feddock C.A. (2007). The lost art of clinical skills. Am. J. Med..

